# Supplementary material for: Content-rich biological network constructed by mining PubMed abstracts
Source: BMC Bioinformatics. 2004 Oct 8;5:147. doi: 10.1186/1471-2105-5-147 (PMC528731; doi:10.1186/1471-2105-5-147)
Supplement: Additional File 2 — The original results of the above study (non-essential files are deleted to keep the file size under the limit set by BMC bioinformatics). [file 1471-2105-5-147-S2.bz2 › chilibotAdditionalFile2/dip05/398ID8663535E9952/html/EGFR_EGF.html]

 


 **EGFR** and **EGF** 
  
Found 3495 abstracts in PubMed, retrieved 05.  
 

 What does Google say? 
 PDF only 
| .edu only 

---

**Interactive relationship** (e.g. stimulation, inhibition, etc)

**Inhibitory relationship**- Co treating PC 3 cells with quercetin significantly attenuated  **EGF**  and TGF alpha induced growth and phosphorylation of ErbB  [ **EGFR** ]  2, ErbB  [ **EGFR** ]  3, c Raf, MAPK kinase 1 2 MEK1 2, MAPK, Elk 1 and Akt 1.  Ref: 12888923 Int J Oncol, 2003
**Neutral relationship**

**Non-interactive relationship** (e.g. studied together, co-existance, homology, etc.)

- Our simulations predict that when  **EGFR**  is activated with TGF alpha, receptor activation is biased toward the cell surface whereas  **EGF**  produces a signaling bias toward the endosomal compartment.  Ref: 12885624 Biophys J, 2003
- The content of urinary bladder cancer antigen UBC, tissue polypeptide specific antigen TPS, nuclear matrix protein 22 NMP22, and the expression of LewisY carbohydrate antigens and of epidermal growth factor receptor  [ **EGFR** ]   **EGF**  R in bladder tumor tissues were determined.  Ref: 12888914 Int J Oncol, 2003
- Inhibition of ErbB  [ **EGFR** ]  2 and ErbB  [ **EGFR** ]  3 expression by quercetin prevents transforming growth factor alpha TGF alpha and epidermal growth factor  **EGF**  induced human PC 3 prostate cancer cell proliferation.  Ref: 12888923 Int J Oncol, 2003
- To determine the utility of this approach, we simulated the differential activation of the  **EGFR**  by two of its ligands, epidermal growth factor  **EGF**  and transforming growth factor alpha TGF alpha.  Ref: 12885624 Biophys J, 2003
